# Impact of pre‐ and post‐diagnosis physical activity on the mortality of patients with cancer: Results from the Health Examinees‐G study in Korea

**DOI:** 10.1002/cam4.6253

**Published:** 2023-06-14

**Authors:** Jaesung Choi, Joo‐Yong Park, Ji‐Eun Kim, Miyoung Lee, Kyuwan Lee, Jong‐Koo Lee, Daehee Kang, Aesun Shin, Ji‐Yeob Choi

**Affiliations:** ^1^ Institute of Health Policy and Management Seoul National University Medical Research Center Seoul South Korea; ^2^ Department of Big Data Medical Convergence Eulji University Seongnam‐Si South Korea; ^3^ Department of Biomedical Sciences Seoul National University Graduate School Seoul South Korea; ^4^ College of Physical Education and Sport Science Kookmin University Seoul South Korea; ^5^ Department of Population Sciences Beckman Research Institute, City of Hope (COH) Duarte California USA; ^6^ Department of Family Medicine College of Medicine Seoul National University Seoul South Korea; ^7^ Department of Preventive Medicine Seoul National University College of Medicine Seoul South Korea; ^8^ Cancer Research Institute Seoul National University Seoul South Korea

**Keywords:** cohort studies, mortality, neoplasms, physical activity

## Abstract

**Background:**

Physical activity (PA) is recommended to improve the survival of cancer patients. However, the prognostic impact of specific PAs is not well understood. Therefore, we investigated the associations of the duration, type, intensity, and number of PAs one participates in pre‐ and post‐diagnosis with mortality in Korean patients with cancer.

**Methods:**

Among the participants aged 40–69 years recruited from the Health Examines study, those diagnosed with cancer after baseline (*n* = 7749) and within 10 years before baseline (*n* = 3008) were included in the analyses for pre‐ and post‐diagnosis PA, respectively. Duration, intensity, type, and number of leisure‐time physical activities participated in were assessed using questionnaires. The Cox proportional hazard model was used to characterize the association between PA and cancer‐specific mortality, adjusting for demographics, behaviors, comorbidities, and cancer stage based on the Surveillance, Epidemiology, and End Results program.

**Results:**

Pre‐diagnosis, patients participating in vigorous‐intensity activities (hazard ratio [HR]: 0.70, 95% confidence interval [CI]: 0.61–0.82), walking (HR: 0.85, 95% CI: 0.74–0.97), climbing (HR: 0.65, 95% CI: 0.55–0.77), sports (HR: 0.39, 95% CI: 0.25–0.61), and more than two activities (HR: 0.73, 95% CI: 0.63–0.86) had significantly lower all‐cause mortality. Importantly, these associations were only found in patients with colorectal cancer participating in vigorous‐intensity activities (HR: 0.40, 95% CI: 0.23–0.70). Post‐diagnosis, only patients who performed more than two activities (HR: 0.65, 95% CI: 0.44–0.95) had significantly lower all‐cause mortality. Similar associations were found for cancer mortality, both pre‐ and post‐diagnosis.

**Conclusion:**

Specific characteristics of PA pre‐ and post‐diagnosis may influence the survival of cancer patients.

## 1 INTRODUCTION

Cancer is the leading cause of death and a challenge for extending life expectancy in most countries worldwide.^1^ In 2020, there were 19.3 million new cancer cases worldwide and nearly 10 million cancer‐related deaths.^2^ According to a report from the World Health Organization, cancer was the leading cause of death before the age of 70 years in 2019.^3^ Although cancer mortality has been declining in Korea since 2002, the number of cancer‐related deaths continues to increase.^4^ The number of cancer‐related deaths was reported to be 81,203 in 2019 (age‐standardized rate: 72.2/100,000).

Physical activity (PA) is known to improve the prognosis of cancer survivors.^5^ Existing exercise guidelines for cancer survivors outline the benefits of PA, including improving anxiety, depressive symptoms, fatigue, quality of life, lymphedema, and physical function.^6^, ^7^ For example, participation in 2–3 combined moderate‐intensity aerobic and resistance exercises per week for at least 12 weeks is effective in enhancing the quality of life. In addition, it has been shown that the optimal timing of PA and specificity may exist. For example, a recent meta‐analysis reported a 20% and 37% decrease in mortality from pre‐ and post‐diagnosis PA, respectively.^8^ However, despite the known benefits of PA for cancer survivors, there is limited evidence of how specific characteristics of PA, such as intensity and type, at different periods are associated with mortality.

Therefore, in this study, we aimed to evaluate the effect of pre‐ and post‐diagnosis PA on all‐cause and cancer‐specific mortality in Korean patients with cancer. In addition, we assessed the association of various characteristics of PA, such as frequency, intensity, time, and type, with mortality pre‐ and post‐diagnosis.

## 2 METHODS

### 2.1 Study population

**FIGURE 1:**
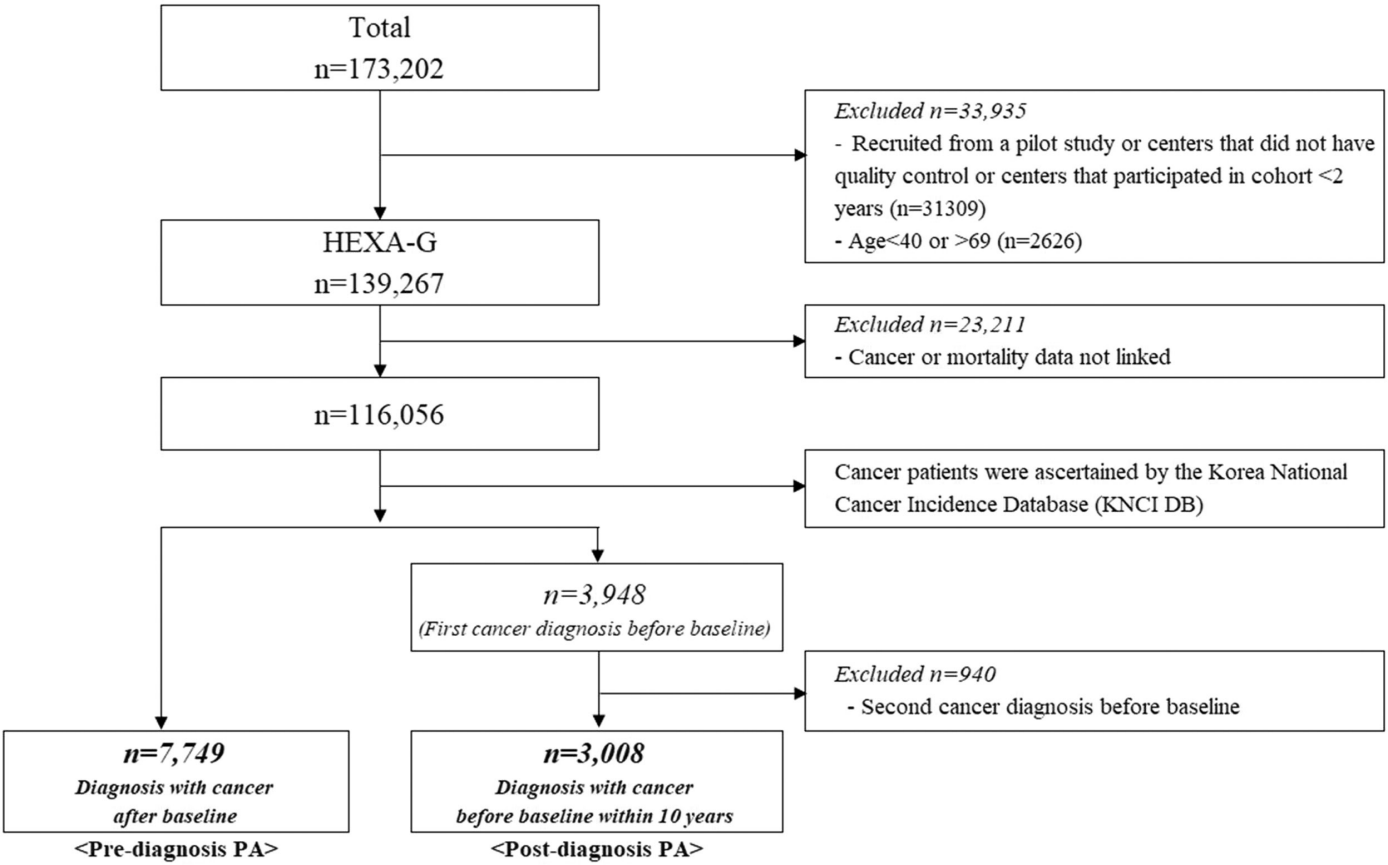
Inclusion and exclusion of the study population.

**FIGURE 2:**
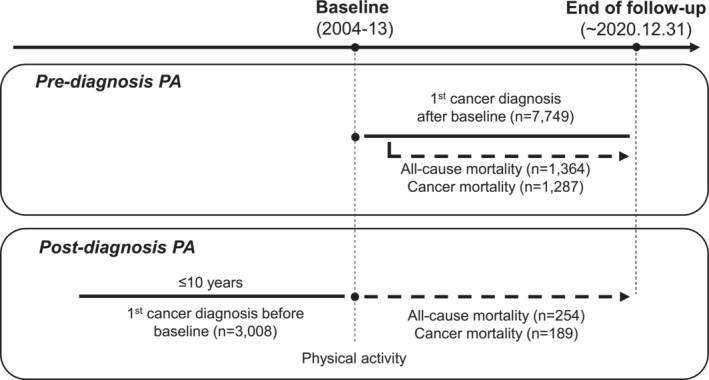
Study design according to pre‐ and post‐diagnosis physical activity.

This longitudinal study was conducted utilizing data from the Health Examinees‐Gem (HEXA‐G) study derived from the Health Examinees study, a component of the Korean Genome and Epidemiology Study (KoGES_HEXA). The characteristics of the cohort have been described previously.^9^, ^10^ Briefly, KoGES_HEXA included participants aged 40–69 years between 2004 and 2013 (*N* = 173,202) from 38 health examination centers and hospitals across the eight regions of Korea. Among the participants of KoGES_HEXA, 139,267 were included in the HEXA‐G study after excluding participants who met the criteria for age and consistency in the recruitment process (Figure 1). Subsequently, 23,211 participants were excluded because their cancer incidence and death data were unavailable. The cancer diagnosis was ascertained using the Korea National Cancer Incidence Database (KNCI DB), compiled by the Korea Central Cancer Registry (KCCR) from 1999 to 2018. Detailed information on the KCCR and KNCI DB has been provided elsewhere.^11^, ^12^ The KCCR database included information on cancer cases, including the date and site of the cancer. Finally, patients diagnosed with cancer after the initial assessment at baseline (*n* = 7749) were included in the analysis of pre‐diagnosis PA. In the post‐diagnosis PA analysis, those diagnosed with cancer before baseline within 10 years (*n* = 3948) were included. Subsequently, those diagnosed with a second cancer before baseline were excluded because their risk of mortality is higher than that of individuals who were not diagnosed with a second cancer.^13^ Finally, 3008 individuals were included in the analysis of post‐diagnosis PA. Those diagnosed with cancer after baseline were included in the study to estimate the association of pre‐diagnosis PA with mortality, while those diagnosed with cancer before baseline were included in the analysis to estimate the association of post‐diagnosis PA with mortality (Figure 2).

All participants voluntarily signed a consent form before participating in the study. The Institutional Review Board of Seoul National University Hospital, Seoul, Korea (IRB Nos. E‐2009‐117‐1159 and E‐2110‐004‐1257) and the ethics committee of the Korean Genome and Epidemiology Study (KoGES) of the Korea National Institute of Health (IRB No. 2014‐08‐02‐3C‐A) approved the study.

### 2.2 Ascertainment of all‐cause mortality

Mortality data were ascertained by linking with the National Death Index from Statistics Korea.^14^ The causes and dates of death were tracked until December 2020. The underlying causes of death were based on the Korean Standard Classification of Diseases codes listed in the National Death Index.^15^ All‐cause mortality included any type of death, including those with specified and unknown causes. Cancer‐specific mortality was defined as the cause of death being coded as malignant (C00‐C97), in situ (D00‐D09), benign (D10‐D36), or unknown (D37‐48).

### 2.3 Assessment of PA

Information on leisure‐time physical activity (LTPA) was collected using self‐reported questionnaires at baseline. First, participants were asked if they had participated in LTPA to the point where they regularly sweat. Participants were then asked about the frequency per week and duration per session. The duration of LTPA per week was calculated by multiplying the duration of each session by the weekly frequency. Participants were then classified based on the recommendations of the international guidelines for PA^16^: as non‐participating (<150 minutes per week) and participating (≥150 minutes per week). Data on participation in 39 common types of activities were collected and classified into six categories based on prior studies (Table S1)^17^, ^18^: walking, climbing, aerobic, recreation, sports, and strengthening activities. Participants were not exclusively classified as participating in each activity category because some participated in more than one activity. The intensity of each activity was determined using the metabolic equivalent (MET) of moderate (<6 MET) and vigorous (≥6 MET) intensity (Table S1).^19^ Subsequently, participants were categorized as non‐participating, participating in only moderate‐intensity LTPA, and participating in vigorous‐intensity LTPA. Diversity of PA was defined as the number of categories one participated in non‐participating, one activity, and more than two activities.

We previously demonstrated the validity and reliability of the KoGES_HEXA PA questionnaire.^20^, ^21^ In brief, LTPA from HEXA was positively correlated with MET‐hours per day using the ActiGraph accelerometer (rho = 0.343, *p* < 0.05), average energy expenditure per day from the multisensor (rho = 0.272, *p* < 0.05), and level of LTPA from the Global Physical Activity Questionnaire (rho = 0.222, *p* < 0.05). The level of PA obtained from HEXA also had moderate to high reliability in repeated measurements at three‐month test–retest intervals (interclass correlation coefficient = 0.722).

### 2.4 Statistical analyses

Using the Cox proportional hazard model, the associations of participation status, duration, intensity, type, and diversity of LTPA with all‐cause mortality were investigated. The list of covariates was selected based on previous literature, and variance inflation factors were estimated to avoid multicollinearity. Finally, the following confounders were included in the model: sex; education (≤middle school, high school, and ≥college); household income in Korean currency (<2 million won, 2–3.9 million won, and ≥4 million won); marital status (living with a spouse or living alone); current occupation (office, manual, and unemployed or housewife); smoking and alcohol consumption status (never, former, and current); body mass index (<18.5, 18.5–24.9, 25.0–29.9, and ≥30 kg/m^2^); average calorie intake (quartile according to sex); self‐reported past diagnoses of hypertension, diabetes, hyperlipidemia, and cardiovascular diseases; and cancer stage based on the Surveillance, Epidemiology, and End Results (SEER) program (I: localized, II: regional, and III: distant). Age was used as the time scale, and person‐time was calculated from the age at cancer diagnosis to the age at death from any cause or the end of follow‐up (December 31, 2020), whichever occurred first. This model was also applied to cancer mortality. In the analyses of cancer mortality, death caused by cancer was defined as an outcome, and death from other causes was censored.

A series of additional analyses were performed. In the analysis of pre‐diagnosis PA, thyroid cancer patients were excluded because of the potential over‐diagnosis of thyroid cancer in Korea in the past decade, particularly in women.^22^ Stratification analyses by SEER stage (Stage I and ≥Stage II) and median value of the time difference between baseline and first cancer occurrence (<5 and ≥5 years) were also performed. A heterogeneity test was conducted to determine whether the associations differed according to the SEER stage and time difference. Moreover, the mortality of type‐specific cancers was evaluated for the most frequently diagnosed cancers, including gastric, colorectal, lung, breast, and prostate cancers. In the analysis of post‐diagnosis PA, mortality in those diagnosed with cancer within 5 years before baseline was evaluated. Sensitivity analysis was also performed after excluding those who were followed up for less than 1 year after baseline to avoid the potential for reverse causation. Stratification analysis was performed using the SEER stage.

## 3 RESULTS

### 3.1 Baseline characteristics

The baseline characteristics of the study population are presented in Table 1. During the follow‐up, 1490 and 254 deaths occurred among the 7749 and 3008 cancer patients diagnosed after baseline (for pre‐diagnosis PA) and before baseline within 10 years (for post‐diagnosis PA), respectively. The median follow‐up duration was 5.12 and 13.48 years for pre‐ and post‐diagnosis PA, respectively. In both groups, women had a decreased risk of all‐cause mortality, while a history of diabetes and SEER stage were associated with an increased risk of all‐cause mortality. In particular, current smoking in the pre‐diagnosis PA group was more likely to be associated with an increased risk of all‐cause mortality than a past diagnosis of diabetes.

**TABLE 1:** Basic characteristics of and hazard ratios for all‐cause mortality in patients diagnosed with cancer after (analysis for pre‐diagnosis physical activity [PA]) and before (analysis for post‐diagnosis PA) the baseline with all‐cause mortality.

	Pre‐diagnosis PA				Post‐diagnosis PA			
	Total	All‐cause			Total	All‐cause		
		Death				Death		
	(%)	(%)	aHR^a^	(95% CI)	(%)	(%)	aHR^a^	(95% CI)
Total (*n*)	7749	1490			3008	254		
Sex								
Men	39.2	57.3	1.00	(ref)	30.2	56.7	1.00	(ref)
Women	60.8	42.7	**0.66**	**(0.55–0.78)**	69.8	43.3	**0.38**	**(0.23–0.63)**
Age								
40–49	26.1	12.3			25.5	15.0		
50–59	38.6	35.6			43.0	29.4		
60–69	35.3	52.1			31.6	55.6		
Education								
≤Middle school	33.3	39.8	1.00	(ref)	33.8	38.5	1.00	(ref)
High school	40.8	38.4	0.98	(0.87–1.11)	43.2	40.1	0.86	(0.60–1.23)
≥College	24.5	20.5	0.89	(0.75–1.05)	21.9	20.3	0.64	(0.40–1.02)
Income per month (10,000 won)								
<200	31.2	40.2	1.00	(ref)	34.8	41.2	1.00	(ref)
200–400	37.3	32.8	**0.79**	**(0.70–0.90)**	36.7	42.2	1.38	(0.98–1.94)
≥4000	21.1	16.4	**0.79**	**(0.66–0.94)**	20.0	10.7	0.74	(0.42–1.28)
Occupation								
Blue collar	31.5	36.6	1.00	(ref)	23.7	21.9	1.00	(ref)
Office job	18.5	16.5	0.99	(0.83–1.17)	14.9	15.0	1.50	(0.89–2.52)
Others^b^	46.8	43.6	0.90	(0.79–1.03)	58.7	60.4	1.35	(0.91–2.00)
Marital status								
Living together	89.6	88.7	1.00	(ref)	89.2	86.1	1.00	(ref)
Living alone	9.7	10.7	**1.27**	**(1.07–1.52)**	10.2	12.8	1.47	(0.94–2.32)
Smoking status								
Never	68.7	52.8	1.00	(ref)	75.0	53.5	1.00	(ref)
Past	17.2	23.6	**1.23**	**(1.04–1.46)**	18.6	36.4	1.45	(0.90–2.34)
Current	13.4	23.3	**1.97**	**(1.67–2.34)**	5.7	9.6	1.26	(0.68–2.35)
Alcohol consumption								
Never	48.5	42.1	1.00	(ref)	61.3	54.5	1.00	(ref)
Past	5.5	7.2	1.08	(0.86–1.37)	11.8	22.5	0.88	(0.58–1.35)
Current	44.5	49.5	0.91	(0.80–1.04)	25.3	21.4	**0.57**	**(0.38–0.86)**
BMI								
<18.5	1.4	1.5	0.89	(0.58–1.37)	2.7	2.1	0.81	(0.30–2.21)
18.5–24.9	63.6	63.0	1.00	(ref)	66.7	68.4	1.00	(ref)
25.0–29.9	31.6	31.8	0.93	(0.83–1.04)	27.8	27.8	0.84	(0.60–1.18)
≥30.0	3.3	3.7	1.22	(0.93–1.62)	2.8	1.6	0.50	(0.15–1.67)
Calorie intake								
1Q	25.7	27.2	1.00	(ref)	27.7	39.0	1.00	(ref)
2Q	24.8	26.9	0.94	(0.82–1.08)	24.8	21.9	**0.64**	**(0.43–0.94)**
3Q	24.2	23.5	0.93	(0.80–1.08)	24.4	20.3	0.68	(0.46–1.01)
4Q	24.1	21.1	**0.78**	**(0.67–0.91)**	22.3	18.7	0.88	(0.58–1.34)
Chronic diseases								
Hypertension								
No	66.4	60.4	1.00	(ref)	70.0	63.6	1.00	(ref)
Yes	33.3	39.5	1.11	(0.99–1.24)	29.8	36.4	1.06	(0.77–1.47)
Diabetes								
No	85.5	79.2	1.00	(ref)	87.4	78.6	1.00	(ref)
Yes	11.1	17.1	**1.39**	**(1.21–1.60)**	10.2	20.3	**1.77**	**(1.21–2.59)**
Hyperlipidemia								
No	54.6	54.6	1.00	(ref)	59.6	63.6	1.00	(ref)
Yes	45.0	45.4	0.90	(0.81–1.00)	40.1	36.4	0.79	(0.58–1.08)
Cardiovascular diseases								
No	95.3	94.2	1.00	(ref)	96.7	92.5	1.00	(ref)
Yes	4.5	5.8	0.81	(0.65–1.02)	3.1	7.5	1.60	(0.91–2.83)
SEER stage								
Localized	53.6	22.1	1.00	(ref)	49.9	39.6	1.00	(ref)
Regional	29.1	29.0	**3.01**	**(2.61–3.48)**	26.7	34.2	**2.06**	**(1.46–2.91)**
Distant	12.1	42.5	**17.37**	**(15.18–19.88)**	3.4	10.7	**4.44**	**(2.67–7.39)**

Bold values for the significant HR and 95%CI.

Abbreviations: aHR, adjusted hazard ratio; BMI, body mass index; CI, confidence interval; SEER, cancer stage based on the Surveillance, Epidemiology, and End Results.

^a^ Hazard ratio adjusted for all variables in the table.

^b^ Unemployed or house wives or career soldier.

### 3.2 Pre‐diagnosis PA

**FIGURE 3:**
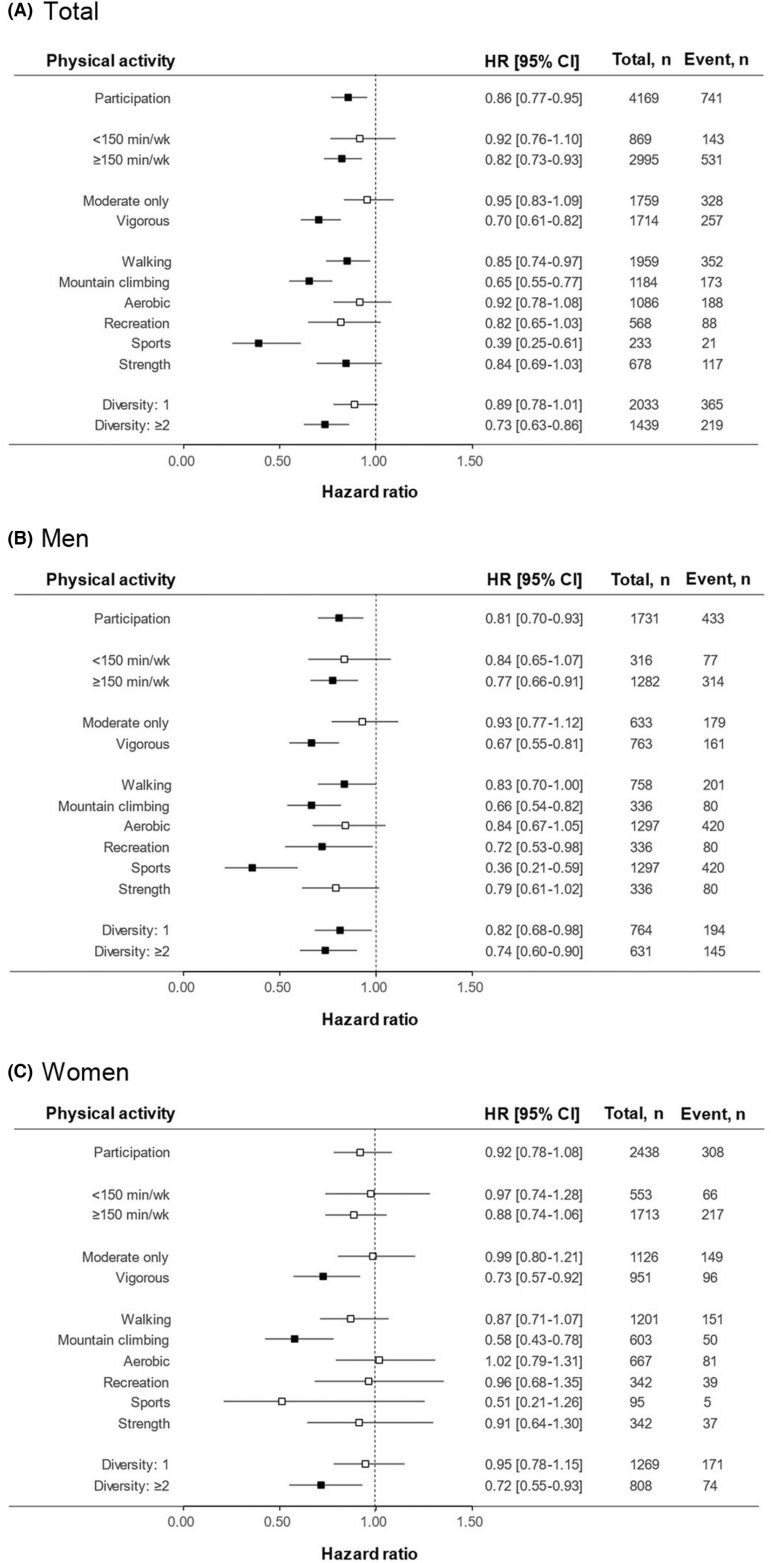
Associations of pre‐diagnosis physical activity (PA) with all‐cause mortality. Forest plot shows the hazard ratios (HRs) and 95% confidence intervals (CIs) adjusted for sex, education, income, occupation, marital status, smoking status, alcohol consumption, body mass index, calorie intake, hypertension, diabetes, hyperlipidemia, cardiovascular diseases, and Surveillance, Epidemiology and End Results stage. The squares represent the HRs, and the horizontal bars extend from the lower limit to the upper limit of the 95% CI of the estimate of the HR. the vertical line indicates “no effect.” (A) Total population; (B) Men; (C) Women.

The association between pre‐diagnosis PA and all‐cause mortality is shown in Figure 3. In the analysis of pre‐diagnosis PA, patients participating in PA for more than 150 minutes/week (hazard ratio [HR]: 0.82, 95% CI: 0.73–0.93), vigorous‐intensity activities (HR: 0.70, 95% CI: 0.61–0.82), walking (HR: 0.85, 95% CI: 0.74–0.97), climbing (HR: 0.65, 95% CI: 0.55–0.77), sports (HR: 0.39, 95% CI: 0.25–0.61), and more than two activities (HR: 0.73, 95% CI: 0.63–0.86) had significantly lower all‐cause mortality. Most of these associations were strengthened in men, whereas only vigorous PA, climbing, and diversity were significantly associated in women. These associations were also consistently found in the analysis of cancer mortality (Figure S1) and after excluding thyroid cancer patients (Figure S2). In addition, the effects of pre‐diagnosis PA were enhanced when the SEER stage was lower (Figure S3) and the period between baseline and first cancer diagnosis was shorter than 5 years (Figure S4). Notably, in the cancer type‐specific analysis (Figure S5), PA duration (HR: 0.54, 95% CI: 0.35–0.82), intensity (HR: 0.40, 95% CI: 0.23–0.70), type (including walking [HR: 0.54, 95% CI: 0.33–0.89], climbing [HR: 0.38, 95% CI: 0.20–0.72], and recreational activities [HR: 0.38, 95% CI: 0.16–0.94]), and diversity (HR: 0.41, 95% CI: 0.22–0.77) were significantly associated with a reduced risk of mortality among colorectal cancer patients. In prostate cancer patients, duration (HR: 0.48, 95% CI: 0.25–0.91) and walking (HR: 0.41, 95% CI: 0.19–0.88) were significantly associated with a reduced risk of mortality. There were no significant associations with other cancer types.

### 3.3 Post‐diagnosis PA

**FIGURE 4:**
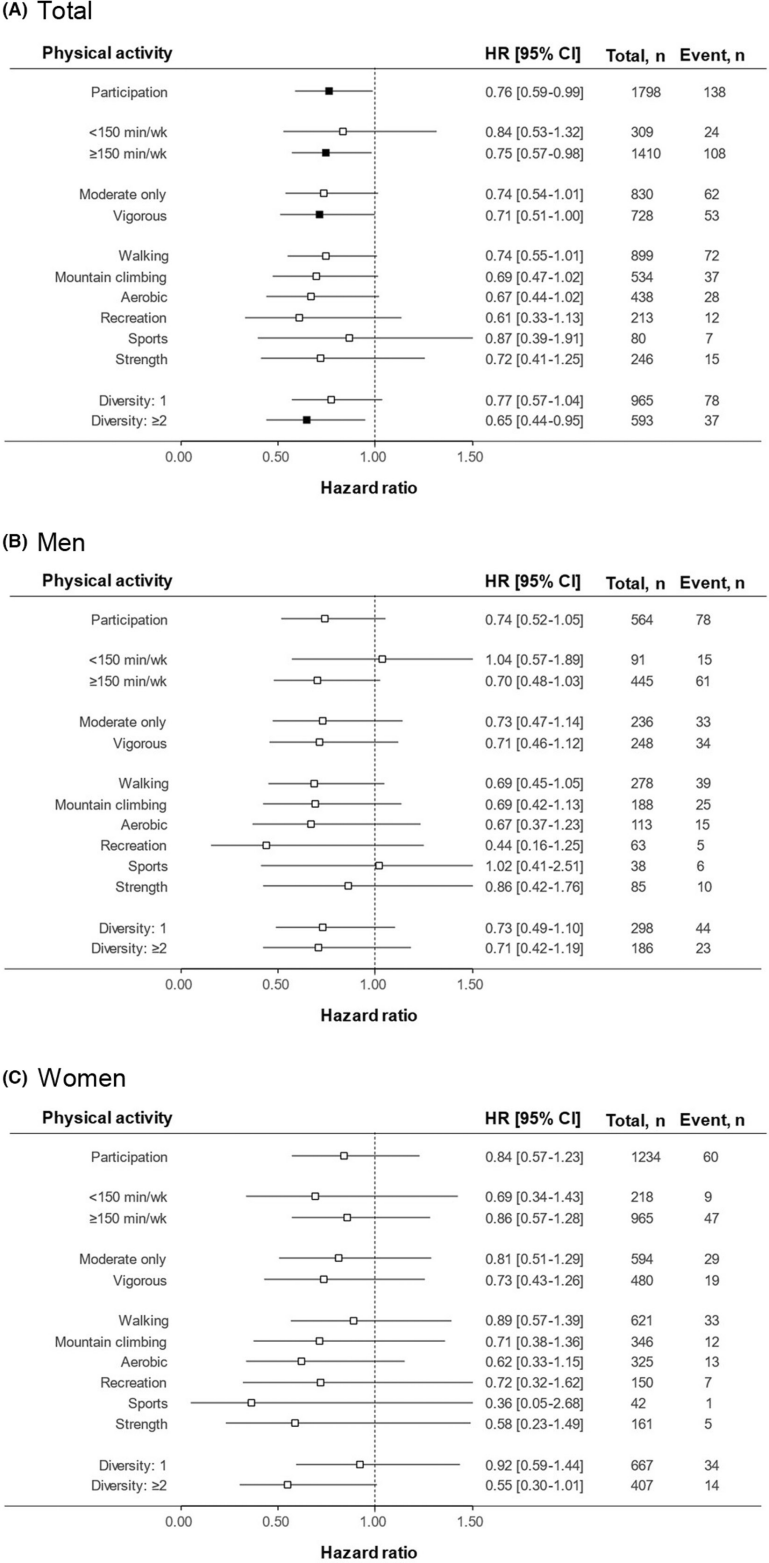
Associations of post‐diagnosis physical activity (PA) with all‐cause mortality. Forest plot shows the hazard ratios (HRs) and 95% confidence intervals (CIs) adjusted for sex, education, income, occupation, marital status, smoking status, alcohol consumption, body mass index, calorie intake, hypertension, diabetes, hyperlipidemia, cardiovascular diseases, and Surveillance, Epidemiology and End Results stage. The squares represent the HRs, and the horizontal bars extend from the lower limit to the upper limit of the 95% CI of the estimate of the HR. the vertical line indicates “no effect.” (A) Total population; (B) Men; (C) Women.

The association between post‐diagnosis PA and all‐cause mortality is shown in Figure 4. Cancer survivors participating in LTPA (HR: 0.76, 95% CI: 0.59–0.99), LTPA for more than 150 minutes/week (HR: 0.75, 95% CI: 0.57–0.98), vigorous‐intensity LTPA (HR: 0.71, 95% CI: 0.51–1.00), and more than two activities (HR: 0.65, 95% CI: 0.44–0.95) had significantly lower all‐cause mortality. Participating in LTPA (HR: 0.71, 95% CI: 0.53–0.91), LTPA for more than 150 minutes/week (HR: 0.71, 95% CI: 0.52–0.97), walking (HR: 0.69, 95% CI: 0.52–0.97), aerobics (HR: 0.60, 95% CI: 0.36–0.99), and more than two activities (HR: 0.57, 95% CI: 0.36–0.90) were associated with significantly lower cancer mortality (Figure S6). In the additional analyses, there were no significant associations between any of the characteristics of LTPA and all‐cause mortality (Figures S7–S9).

## 4 DISCUSSION

In this study, participation in and duration, intensity, type, and diversity of pre‐diagnosis LTPA were associated with a lower risk of all‐cause and cancer mortality. In particular, vigorous intensity, mountain climbing, and participating in more than two types of activities were strongly associated with all‐cause and cancer mortality in both men and women. These associations were strengthened when patients with cancer were defined in a shorter period. Moreover, these associations were found only in patients with colorectal cancer. Post‐diagnosis, participation, duration, intensity, and diversity were associated with all‐cause and cancer mortality.

A recent meta‐analysis of 33 prospective cohort studies calculated a pooled relative risk of cancer mortality of 0.82 (95% CI: 0.79–0.86) for the highest versus lowest pre‐diagnosis PA levels in overall cancer survivors.^8^ Previous studies also reported that participation in general sports activities such as cycling, swimming, running, aerobics, dancing, and ball sports such as football and tennis were most strongly associated with a 53% reduction in mortality risk.^23^ Our findings on pre‐diagnosis LTPA confirm previous findings that various types of LTPA, such as walking for pleasure, mountain climbing, and sports, are associated with a lower risk of all‐cause and cancer mortality. Furthermore, we highlight the importance of LTPA intensity, the number of activity types participated in, and the strong short‐term effects of LTPA. Maintaining a regular exercise regimen before a cancer diagnosis, especially vigorous and diverse activities, is important for public health because it could reduce the risk of cancer and simultaneously improve the prognosis of recently diagnosed cancer.

Regarding post‐diagnosis PA, a recent meta‐analysis of four prospective cohort studies calculated that the highest versus lowest levels of post‐diagnosis PA were associated with a lower risk of mortality (0.63, 95% CI: 0.53–0.75) in overall cancer survivors.^8^ In our study, the evidence could be expanded so that the number of LTPAs one participated in would be considered the strongest prognostic factor, especially after a cancer diagnosis. Furthermore, these results can be used as a basis to explain how exercise modalities play a role in cancer progression in Asians.^24^

To the best of our knowledge, this is the first study to reveal the protective effects of pre‐diagnosis PA on colorectal cancer mortality in an Asian population. In addition, unlike previous studies, we suggest an optimal modality of PA in which intensity is the most significant characteristic pre‐diagnosis for the secondary prevention of colorectal cancer. In addition, considering that mountain climbing, which also has a long duration, is the main source of vigorous‐intensity LTPA, a longer duration of vigorous‐intensity PA would be the most significant characteristic of LTPA in Asian cancer patients, like in Caucasian colorectal cancer patients.^25^ There is abundant evidence for the protective effect of PA on the mortality of colorectal cancer survivors in different periods.^26^ For example, pre‐ and post‐diagnosis PA are reported to be associated with a lower risk of all‐cause mortality.^27^, ^28^, ^29^ However, only two studies reported on the protective effects of post‐diagnosis PA in the Asian population, especially in Korea,^30^, ^31^ and no studies have addressed pre‐diagnosis PA. Thus, the importance of vigorous‐intensity pre‐diagnosis PA should be considered when formulating policies targeted at the primary and secondary prevention of colorectal cancer.

Several biological mechanisms have been proposed to explain the association between PA and mortality. PA may mediate numerous pathways involved in cancer progression and the development of other chronic diseases, including energy metabolism, insulin, chronic inflammation, the immune system, and antioxidant pathways.^32^, ^33^, ^34^, ^35^ Another proposed mechanism is that PA alters adipocytokine levels, which may affect cancer morbidity and mortality by decreasing inflammatory adipocytokines and increasing anti‐inflammatory cytokines.^36^ In addition, PA can improve cardiovascular function by effectively controlling high blood pressure.^37^ The Physical Exercise Across the Cancer Experience framework, which describes the effect of PA according to the periods before and after diagnosis, proposes an advantage of PA in enhancing the cancer treatment effect in the treatment stage and improving overall health and immune function after treatment.^38^

The strengths of our study include its prospective design, adjustment for several important confounders, and the availability of information on PA. Although several studies have been conducted in Asia^39^, ^40^, ^41^, ^42^, ^43^, ^44^, ^45^, ^46^, ^47^, ^48^, ^49^ with the general population, we only included patients with cancer in the analysis. In addition, we utilized population‐based cohorts by linking them with representative national cancer and death registries, allowing pre‐ and post‐diagnosis analyses to be performed within the same cohort. Finally, the PA variables used in this study included various characteristics, including duration, intensity, type, and diversity.

Nevertheless, several limitations should be considered when interpreting our findings. First, the study populations for pre‐ and post‐diagnosis PA were not identical, so the characteristics of each study group were slightly different. For example, there was a higher proportion of women in the post‐diagnosis PA group; thus, there were more housewives and past consumers of alcohol (a trait more commonly found among women in Korea) in the group. However, the age structure and level of PA were similar between the two groups. Additionally, because those diagnosed with cancer before baseline were cancer survivors, healthier and milder cases were included in the analysis. This could have impacted the results moving toward the null. Another limitation stemming from the non‐identical patient groups is the difference in statistical power. The number of patients included in the post‐diagnosis analysis was less than those in the pre‐diagnosis analysis; this may explain why more of the associations reached statistical significance in the pre‐diagnosis group and should be confirmed in future studies. Second, information on PA was not measured repeatedly during follow‐up. Thus, we cannot exclude the possibility that changes in PA behavior over time influenced our results. Third, the study lacked sufficient power to examine specific cancer sites in the analysis of post‐diagnosis PA. Furthermore, since most deaths were due to cancer, resulting in consistent associations between all‐cause and cancer‐specific mortality, we could not assess the risk of non‐cancer mortality, such as CVD or accidental mortality. For example, since patients with cancer have a lower level of physical function, PA is known to have a protective effect against accidental mortality. In future studies, the risk of mortality due to causes other than cancer should be evaluated according to different periods to determine the importance of PA. Finally, no information regarding cancer treatment was available. However, we adjusted for the tumor stage at diagnosis. As cancer treatment generally follows the tumor stage, the stage can be considered a valid surrogate variable for cancer treatment, with rare exceptions.

## 5 CONCLUSION

In this population‐based cohort study of cancer survivors, we found that pre‐diagnosis PA, including vigorous‐intensity activity, was strongly associated with a lower risk of all‐cause and cancer mortality in the Asian population. Notably, these associations were only found in patients with colorectal cancer. Post‐diagnosis, the duration and diversity of LTPA had a protective effect on all‐cause and cancer‐related mortality. This result highlights the considerable significance of LTPA in decreasing the risk of mortality both pre‐ and post‐diagnosis. Regarding public health recommendations, our findings show that engagement in vigorous‐intensity PA before diagnosis strongly reduces the risk of mortality in cancer survivors. These results suggest the need for greater public awareness of the protective effects of PA on the survival of cancer patients, and future randomized controlled trials are warranted to demonstrate the impact of PA on cancer patients.

## AUTHOR CONTRIBUTIONS

**Jaesung Choi:** Conceptualization (equal); data curation (equal); formal analysis (equal); investigation (equal); methodology (equal); resources (equal); software (equal); visualization (equal); writing – original draft (equal). **Joo‐Yong Park:** Formal analysis (equal); methodology (equal); visualization (equal); writing – review and editing (equal). **Ji‐Eun Kim:** Formal analysis (equal); methodology (equal); visualization (equal); writing – review and editing (equal). **Miyoung Lee:** Conceptualization (equal); methodology (equal); visualization (equal); writing – review and editing (equal). **Kyuwan Lee:** Conceptualization (equal); methodology (equal); visualization (equal); writing – review and editing (equal). **Jong‐koo Lee:** Conceptualization (equal); methodology (equal); visualization (equal); writing – review and editing (equal). **Daehee Kang:** Conceptualization (equal); methodology (equal); visualization (equal); writing – review and editing (equal). **Aesun Shin:** Conceptualization (equal); methodology (equal); visualization (equal); writing – review and editing (equal). **Ji‐Yeob Choi:** Conceptualization (lead); formal analysis (supporting); funding acquisition (lead); methodology (lead); project administration (lead); resources (equal); supervision (lead); visualization (equal); writing – original draft (equal).

## FUNDING INFORMATION

This work was funded by Korea Centers for Disease Control and Prevention (2004‐E71004‐00, 2005‐E71011‐00, 2005‐E71009‐00, 2006‐E71001‐00, 2006‐E71004‐00, 2006‐E71010‐00, 2006‐E71003‐00, 2007‐E71004‐00, 2007‐E71006‐00, 2008‐E71006‐00, 2008‐E71008‐00, 2009‐E71009‐00, 2010‐E71006‐00, 2011‐E71006‐00, 2012‐E71001‐00, 2013‐E71009‐00, 2018‐P7106‐00, and 2021‐E0608‐00) and the Ministry of Education of the Republic of Korea and the National Research Foundation of Korea (NRF‐2021R1I1A1A01052952 and NRF‐2022R1A2B5B01002471).

## CONFLICT OF INTEREST STATEMENT

All authors declare no support from any organization for the submitted work.

### ETHICS STATEMENT

All participants voluntarily signed a consent form before participating in the study. The Institutional Review Board of Seoul National University Hospital, Seoul, Korea (IRB Nos. E‐2009‐117‐1159 and E‐2110‐004‐1257) and the ethics committee of the Korean Genome and Epidemiology Study (KoGES) of the Korea National Institute of Health (IRB No. 2014‐08‐02‐3C‐A) approved the study.

## Supporting information


Figure S1–S9.

Table S1.

## Data Availability

Approval of the KoGES data is available through https://nih.go.kr/contents.es?mid=a40504060100. NIH permits access to all of these data via download for any researcher who promises to follow the research ethics.
