# Supplementary material for: Impact of pre‐ and post‐diagnosis physical activity on the mortality of patients with cancer: Results from the Health Examinees‐G study in Korea
Source: Cancer Med. 2023 Jun 14;12(15):16591–603. doi: 10.1002/cam4.6253 (PMC10469756; doi:10.1002/cam4.6253)
Supplement: Supplementary file 1 — Figure S1–S9. [file CAM4-12-16591-s002.pdf]

(A) Total

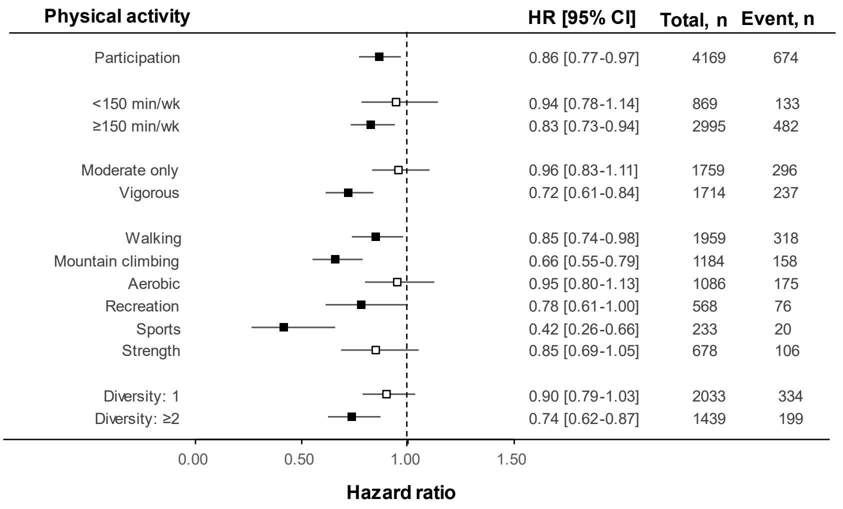

(B) Men

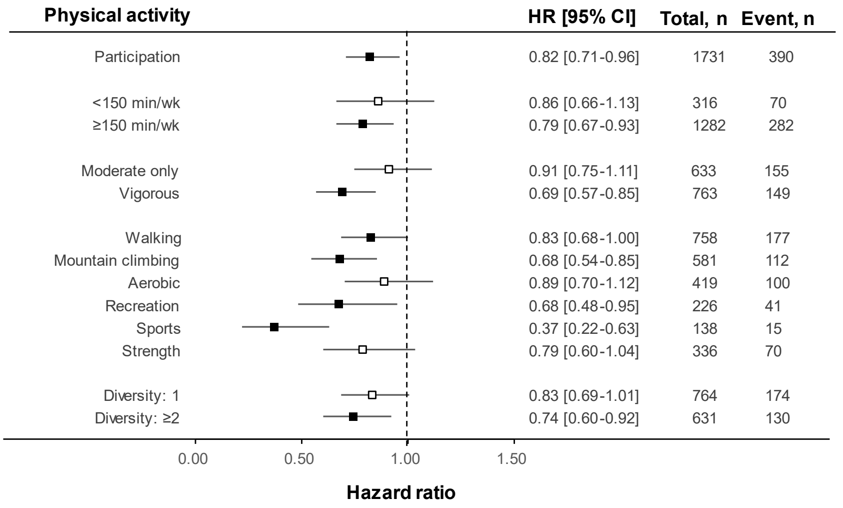

(C) Women

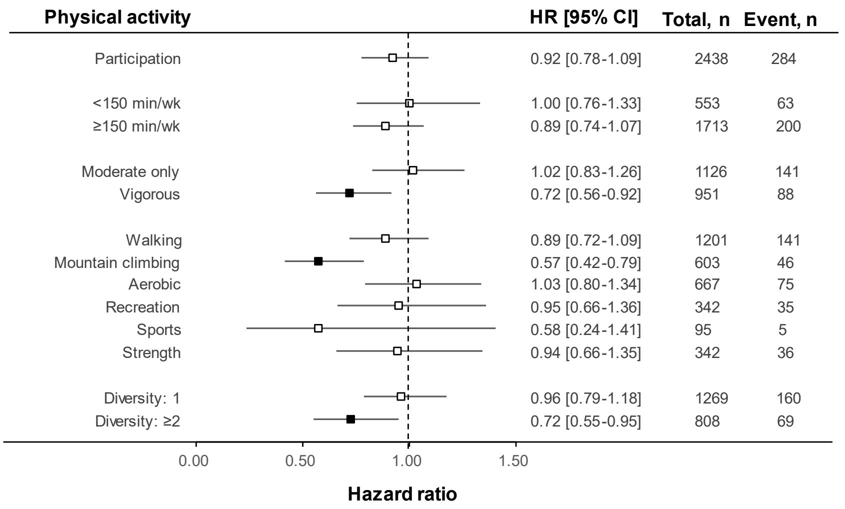

Supplementary Figure 1. Associations of pre-diagnosis physical activity with cancer mortality

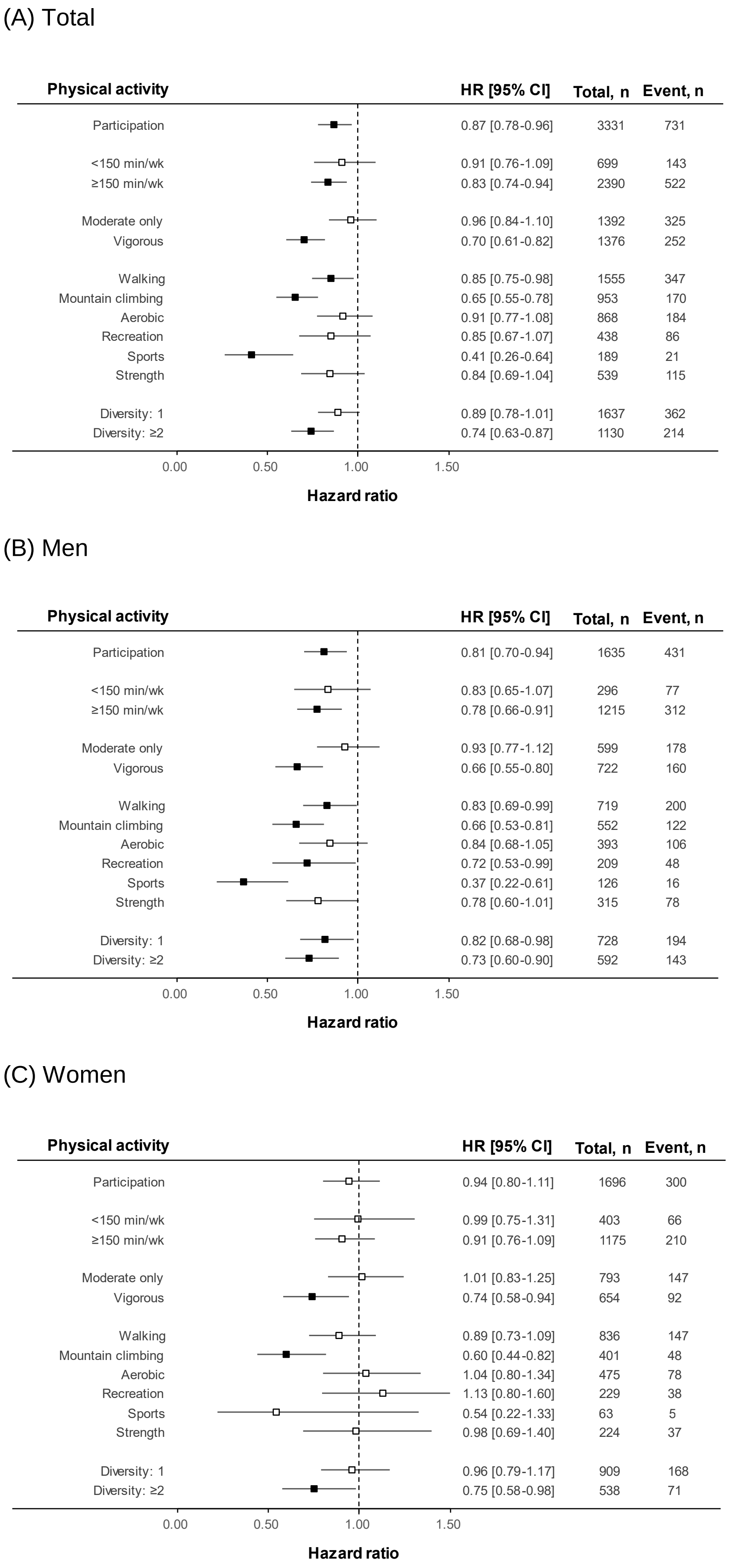

Supplementary Figure 2. Associations of pre-diagnosis physical activity with all-cause mortality excluding thyroid cancer patients

(A) Total

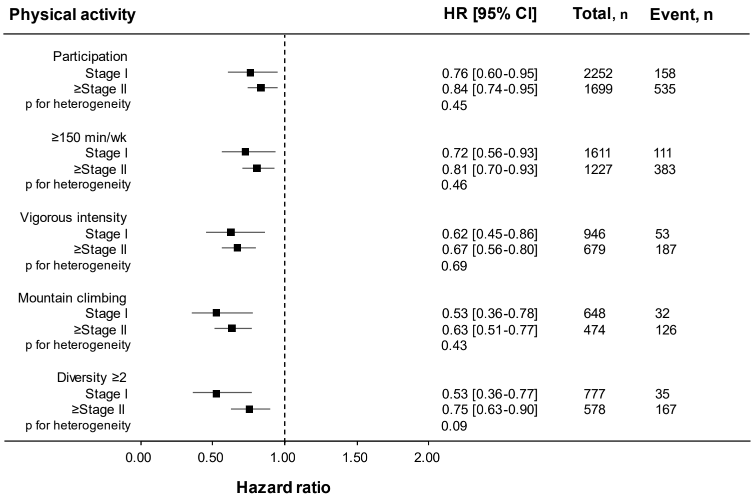

(B) Men

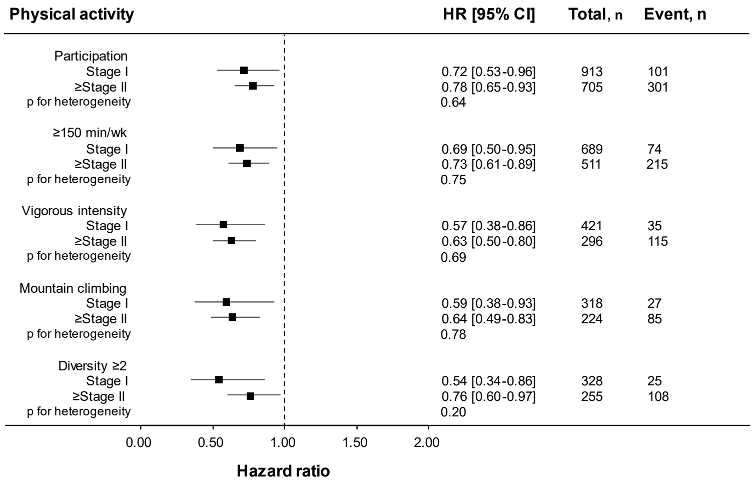

(C) Women

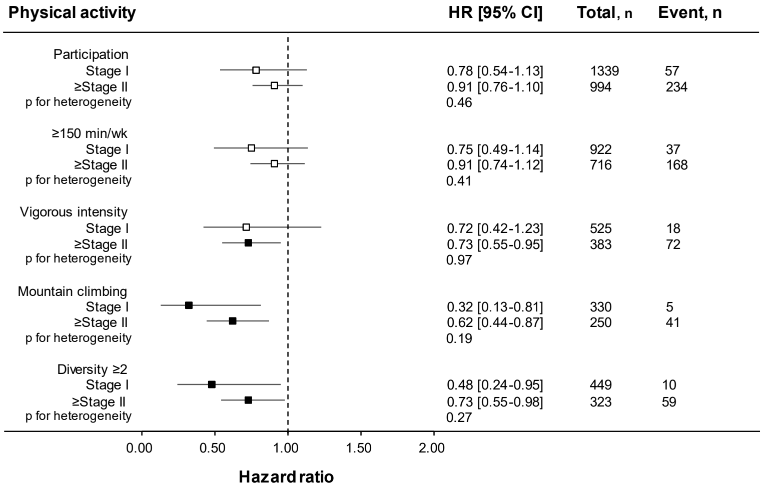

Supplementary Figure 3. Associations of pre-diagnosis physical activity with all-cause mortality according to SEER stage

(A) Total

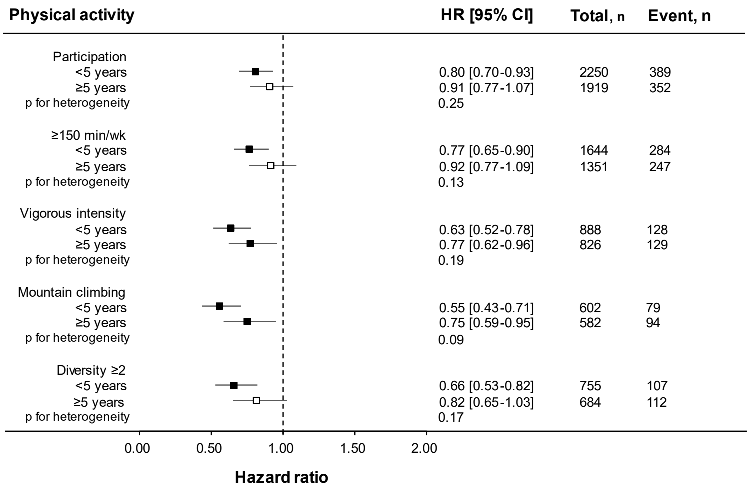

(B) Men

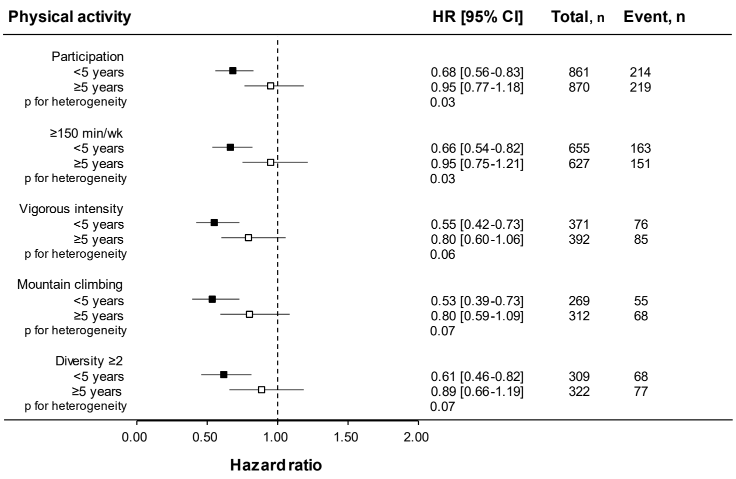

(C) Women

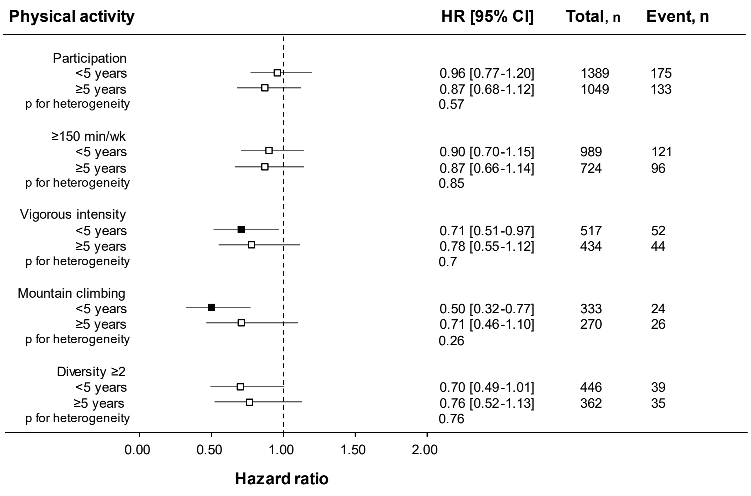

Supplementary Figure 4. Associations of pre-diagnosis physical activity with all-cause mortality according to the period between baseline and first cancer diagnosis

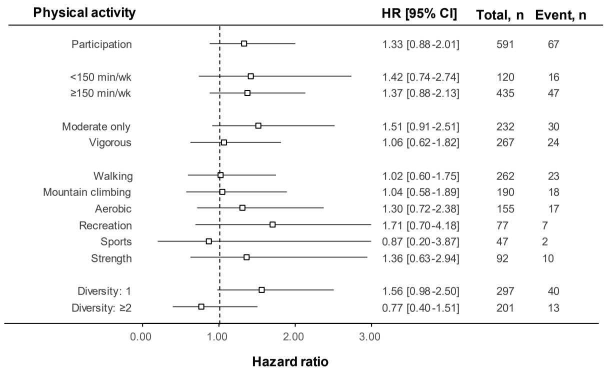

(A) Gastric cancer

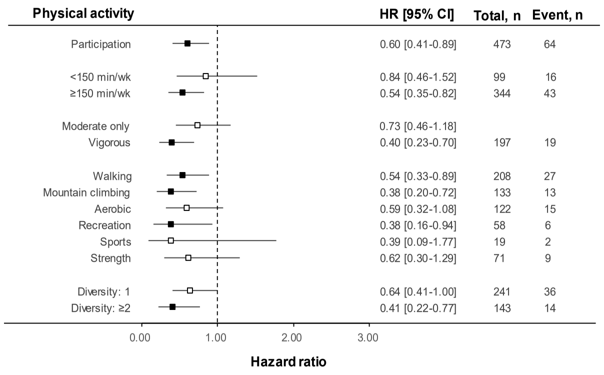

(B) Colorectal cancer

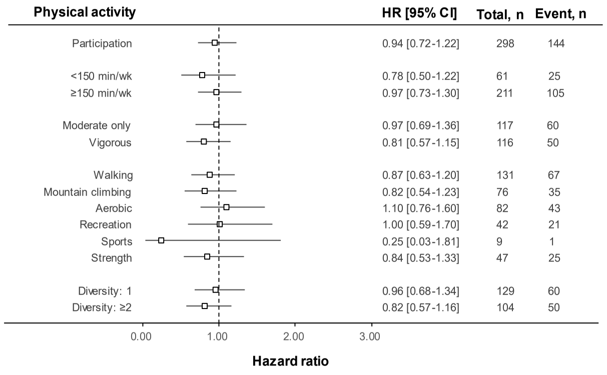

(C) Lung cancer

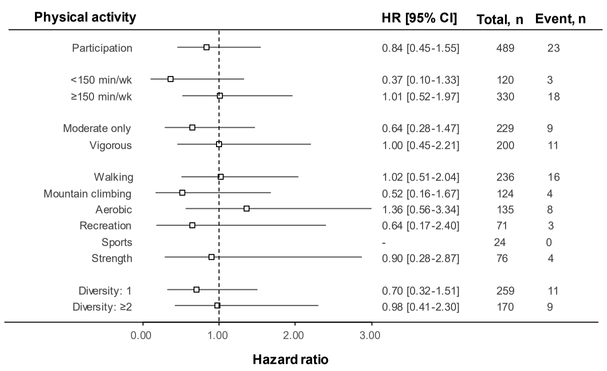

(D) Breast cancer

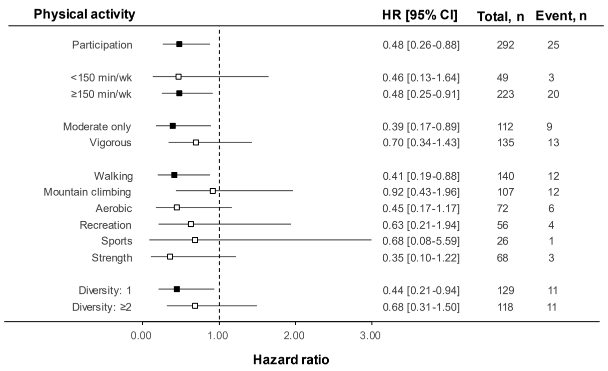

(E) Prostate cancer

Supplementary Figure 5. Associations of pre-diagnosis physical activity with all-cause mortality according to cancer type

(A) Total

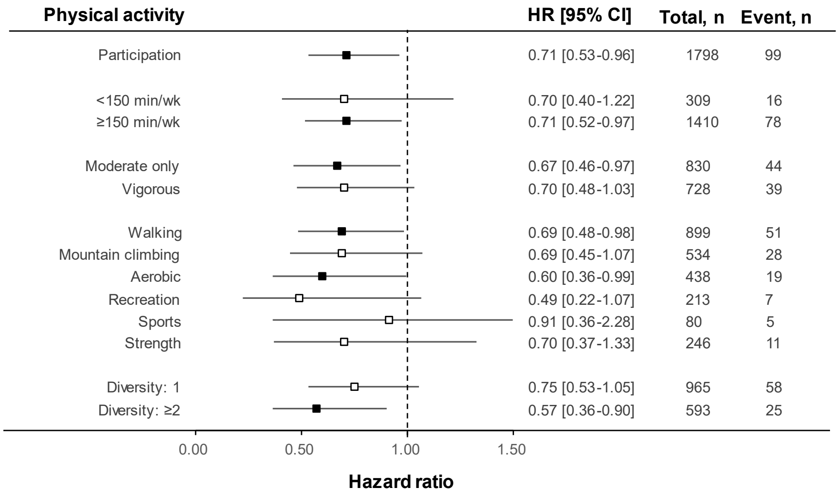

Physical activity	HR [95% CI]	Total, n	Event, n
Participation	0.71 [0.53-0.96]	1798	99
<150 min/wk	0.70 [0.40-1.22]	309	16
≥150 min/wk	0.71 [0.52-0.97]	1410	78
Moderate only	0.67 [0.46-0.97]	830	44
Vigorous	0.70 [0.48-1.03]	728	39
Walking	0.69 [0.48-0.98]	899	51
Mountain climbing	0.69 [0.45-1.07]	534	28
Aerobic	0.60 [0.36-0.99]	438	19
Recreation	0.49 [0.22-1.07]	213	7
Sports	0.91 [0.36-2.28]	80	5
Strength	0.70 [0.37-1.33]	246	11
Diversity: 1	0.75 [0.53-1.05]	965	58
Diversity: ≥2	0.57 [0.36-0.90]	593	25

(B) Men

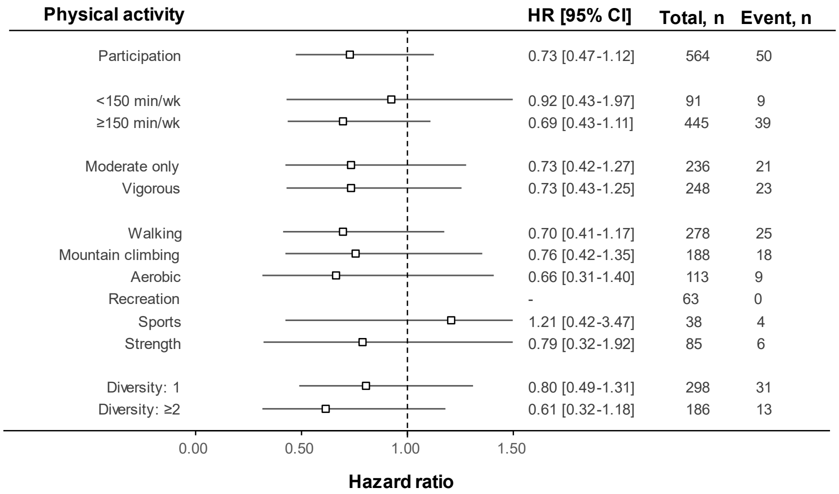

Physical activity	HR [95% CI]	Total, n	Event, n
Participation	0.73 [0.47-1.12]	564	50
<150 min/wk	0.92 [0.43-1.97]	91	9
≥150 min/wk	0.69 [0.43-1.11]	445	39
Moderate only	0.73 [0.42-1.27]	236	21
Vigorous	0.73 [0.43-1.25]	248	23
Walking	0.70 [0.41-1.17]	278	25
Mountain climbing	0.76 [0.42-1.35]	188	18
Aerobic	0.66 [0.31-1.40]	113	9
Recreation	-	63	0
Sports	1.21 [0.42-3.47]	38	4
Strength	0.79 [0.32-1.92]	85	6
Diversity: 1	0.80 [0.49-1.31]	298	31
Diversity: ≥2	0.61 [0.32-1.18]	186	13

(C) Women

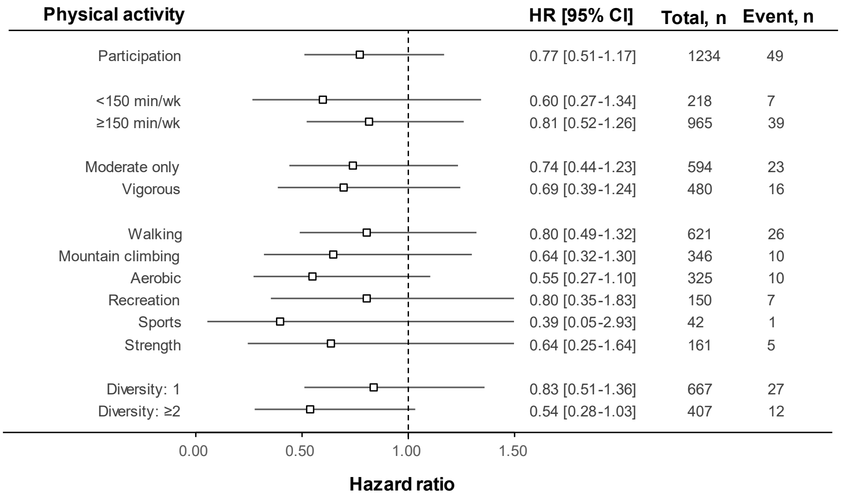

Physical activity	HR [95% CI]	Total, n	Event, n
Participation	0.77 [0.51-1.17]	1234	49
<150 min/wk	0.60 [0.27-1.34]	218	7
≥150 min/wk	0.81 [0.52-1.26]	965	39
Moderate only	0.74 [0.44-1.23]	594	23
Vigorous	0.69 [0.39-1.24]	480	16
Walking	0.80 [0.49-1.32]	621	26
Mountain climbing	0.64 [0.32-1.30]	346	10
Aerobic	0.55 [0.27-1.10]	325	10
Recreation	0.80 [0.35-1.83]	150	7
Sports	0.39 [0.05-2.93]	42	1
Strength	0.64 [0.25-1.64]	161	5
Diversity: 1	0.83 [0.51-1.36]	667	27
Diversity: ≥2	0.54 [0.28-1.03]	407	12

Supplementary Figure 6. Associations of post-diagnosis physical activity with cancer mortality

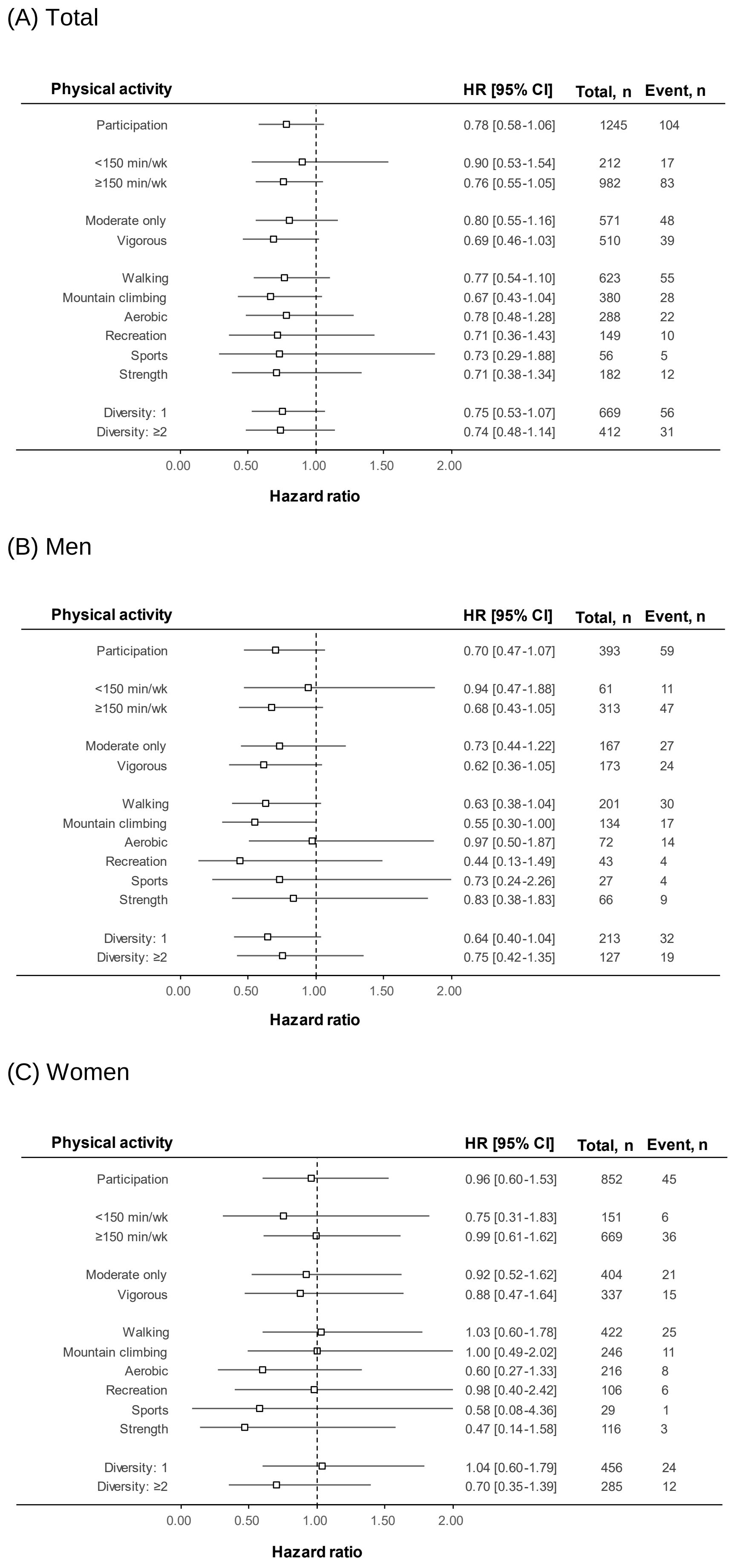

Supplementary Figure 7. Associations of post-diagnosis physical activity with all-cause mortality in cancer patients diagnosed within five years before baseline

(A) Total

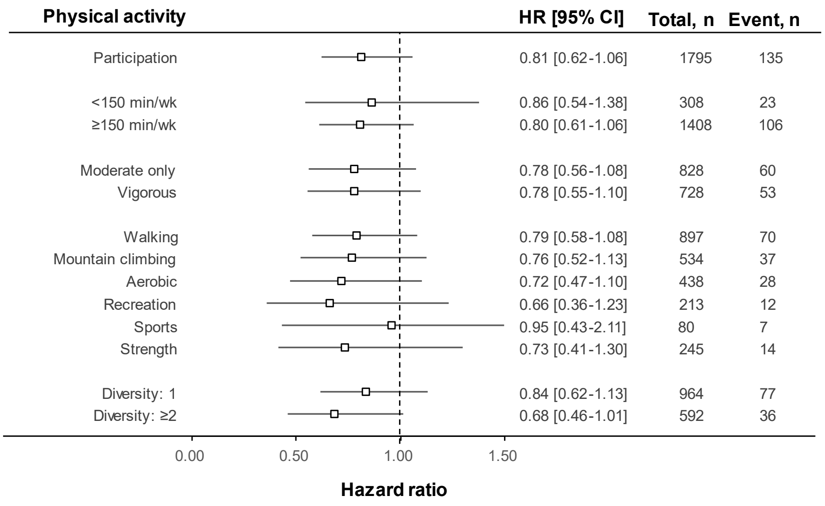

(B) Men

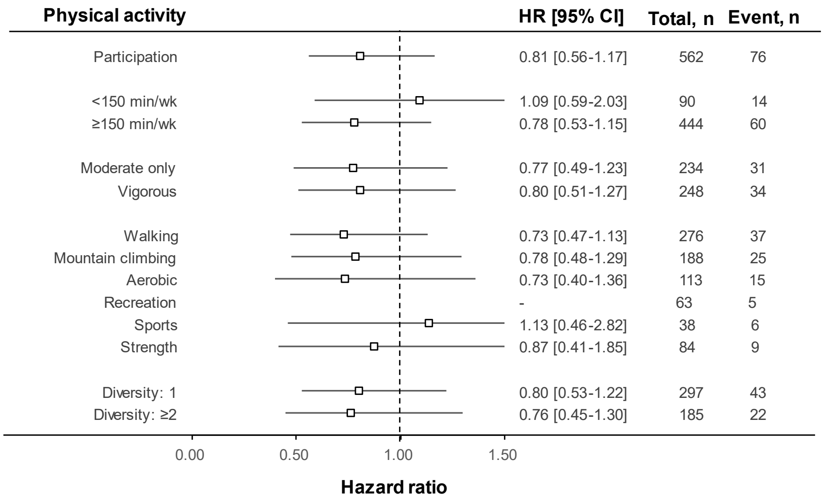

(C) Women

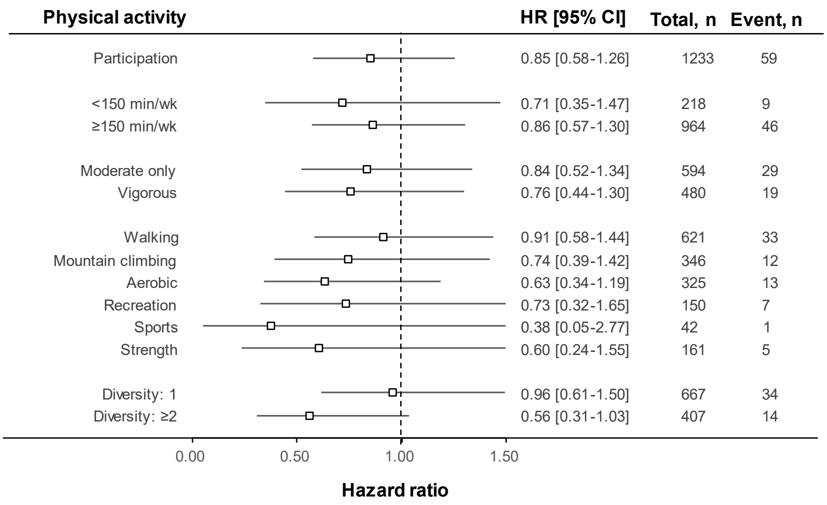

Supplementary Figure 8. Associations of post-diagnosis physical activity with all-cause mortality after excluding those who were followed up for less than one year after baseline

(A) Total

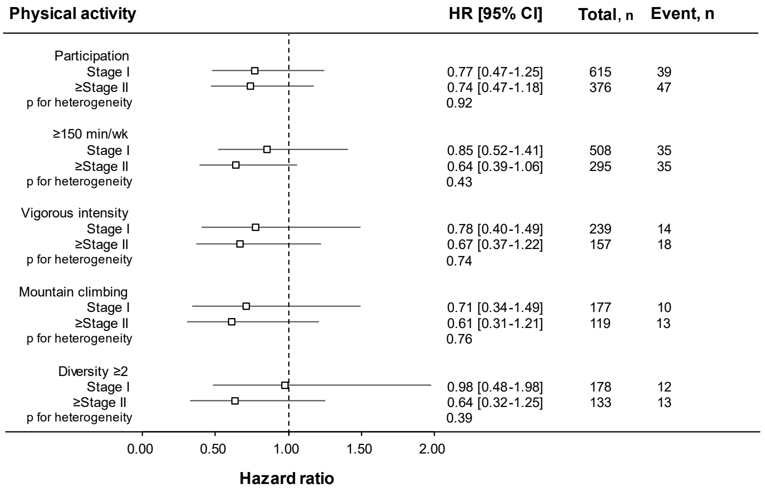

(B) Men

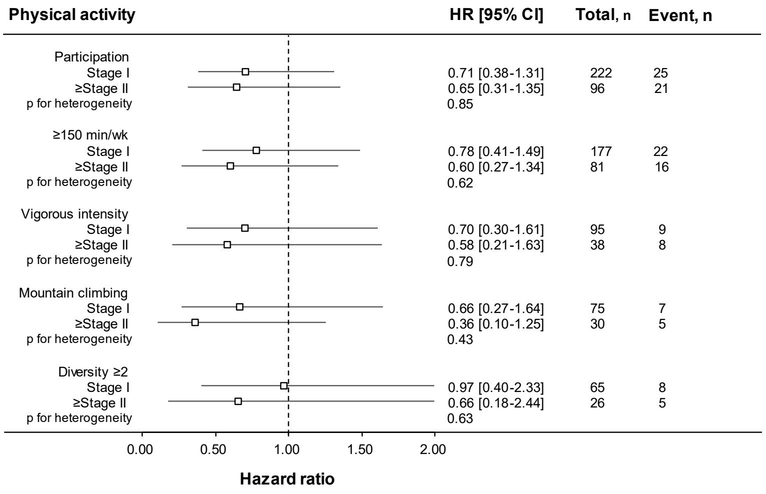

(C) Women

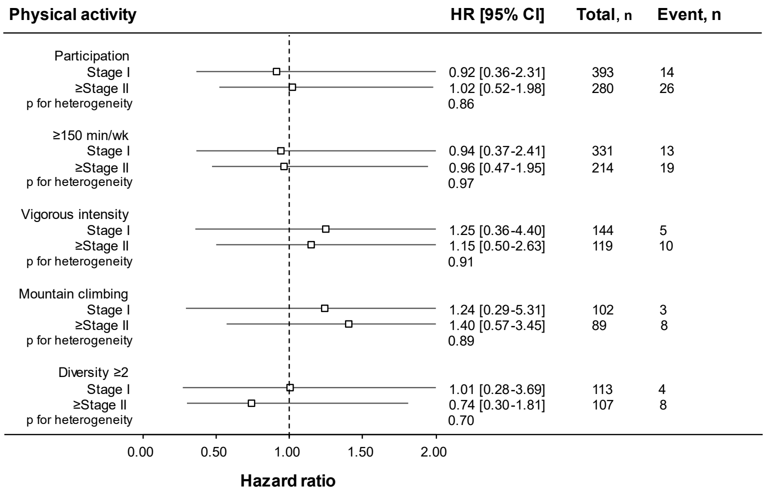

Supplementary Figure 9. Associations of post-diagnosis physical activity with all-cause mortality according to SEER stage
