# Supplementary material for: Impact of pre‐ and post‐diagnosis physical activity on the mortality of patients with cancer: Results from the Health Examinees‐G study in Korea
Source: Cancer Med. 2023 Jun 14;12(15):16591–603. doi: 10.1002/cam4.6253 (PMC10469756; doi:10.1002/cam4.6253)
Supplement: Supplementary file 2 — Table S1. [file CAM4-12-16591-s001.docx]

**Supplementary Table 1.** Categories and activities included in each category of leisure-time physical activity

| Category | Included activities | Reference for types^1^ | Intensity^2^ |
| --- | --- | --- | --- |
| Leisure-time walk | Leisure-time walk | ADA; Chekroud et al. | Moderate |
| Mountain climbing | Climb | ADA; Chekroud et al. | Vigorous |
| Aerobic & Gym | Jump | ADA; Chekroud et al. | Moderate |
|  | Fast-walk | ADA; Chekroud et al. | Moderate |
|  | Gymnastic | ADA; Chekroud et al. | Moderate |
|  | Aerobic | ADA; Chekroud et al. | Vigorous |
|  | Bicycle | ADA; Chekroud et al. | Vigorous |
|  | Dance | ADA; Chekroud et al. | Vigorous |
|  | Jogging | ADA; Chekroud et al. | Vigorous |
|  | In-line skate | ADA; Chekroud et al. | Vigorous |
|  | Jump rope | ADA; Chekroud et al. | Vigorous |
|  | Skate | ADA; Chekroud et al. | Vigorous |
|  | Swim | ADA; Chekroud et al. | Vigorous |
|  | Judo | Chekroud et al. | Vigorous |
| Recreation | Bending | Chekroud et al. | - |
|  | Shoot | Chekroud et al. | Light |
|  | Yoga | Chekroud et al. | Moderate |
|  | Bowling | Chekroud et al. | Moderate |
|  | Fishing | Chekroud et al. | Moderate |
|  | Golf | Chekroud et al. | Moderate |
|  | Indoor golf | Chekroud et al. | Moderate |
|  | Billiard | Chekroud et al. | Moderate |
|  | Gate ball | Chekroud et al. | Moderate |
|  | Horse ride | Chekroud et al. | Moderate |
|  | Ski | Chekroud et al. | Vigorous |
|  | Sports climbing | Chekroud et al. | Vigorous |
| Sports (team-based) | Badminton | Chekroud et al. | Moderate |
|  | Ping-pong | Chekroud et al. | Moderate |
|  | Baseball | Chekroud et al. | Moderate |
|  | Racket ball | Chekroud et al. | Vigorous |
|  | Tennis | Chekroud et al. | Vigorous |
|  | Basket ball | Chekroud et al. | Vigorous |
|  | Soccer | Chekroud et al. | Vigorous |
| Strength | Dumbbell | ADA | Moderate |
|  | Fitness center | ADA | Moderate |
|  | Muscle exercise | ADA | Moderate |
|  | Push-up | ADA | Moderate |
|  | Leapfrog | ADA | Moderate |
|  | Sit-up | ADA | Moderate |

^1^ADA, American Diabetes Association (http://www.diabetes.org/food-and-fitness/fitness/types-of-activity/); Chekroud et al. *Lancet Psychiatry* 2018

^2^Ainsworth BE et al. *Medicine and Science in Sports and Exercise* 2011
